# Supplementary material for: The solution structure of Dead End bound to AU-rich RNA reveals an unusual mode of tandem RRM-RNA recognition required for mRNA regulation
Source: Nat Commun. 2022 Oct 6;13:5892. doi: 10.1038/s41467-022-33552-x (PMC9537309; doi:10.1038/s41467-022-33552-x)
Supplement: Supplementary file 1 — Supplementary Information [file 41467_2022_33552_MOESM1_ESM.pdf]

Supplementary Information

The solution structure of Dead End bound to AU-rich RNA reveals an unusual mode of tandem RRM-RNA recognition required for mRNA regulation

Authors

Malgorzata M. Duszczyk, Harry Wischnewski, Tamara Kazeeva, Rajika Arora, Fionna E. Loughlin, Christine von Schroetter, Ugo Pradère, Jonathan Hall, Constance Ciaudo, Frédéric H.-T. Allain

Supplementary Figure 1

A

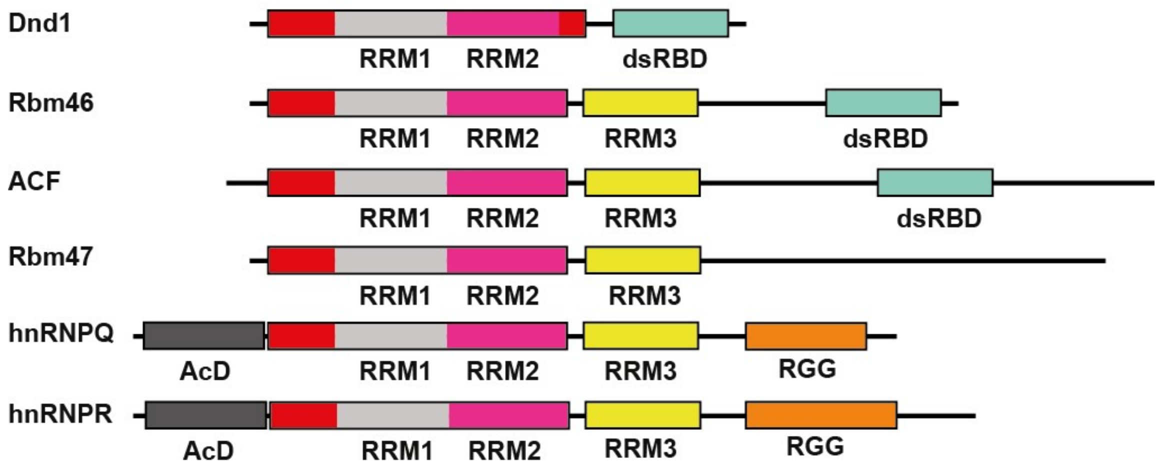

B

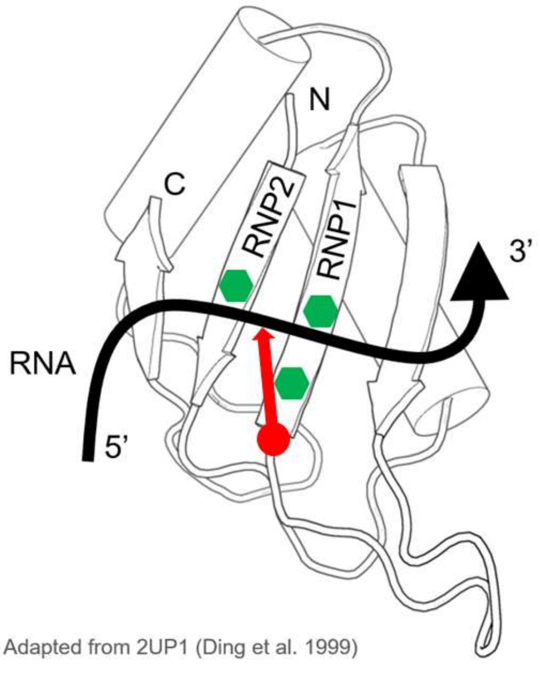

RNP1 on β3 [R/K]-G-[F/Y]-[G/A]-[F/Y]-[I/L/V]-X-[F/Y] DND1:R98-G-F100-A-Y102-A-R-Y

RNP2 on β1 [I/L/V]-[F/Y]-[I/L/V]-X-N-L DND1: V-F61-I-G-R-L

C

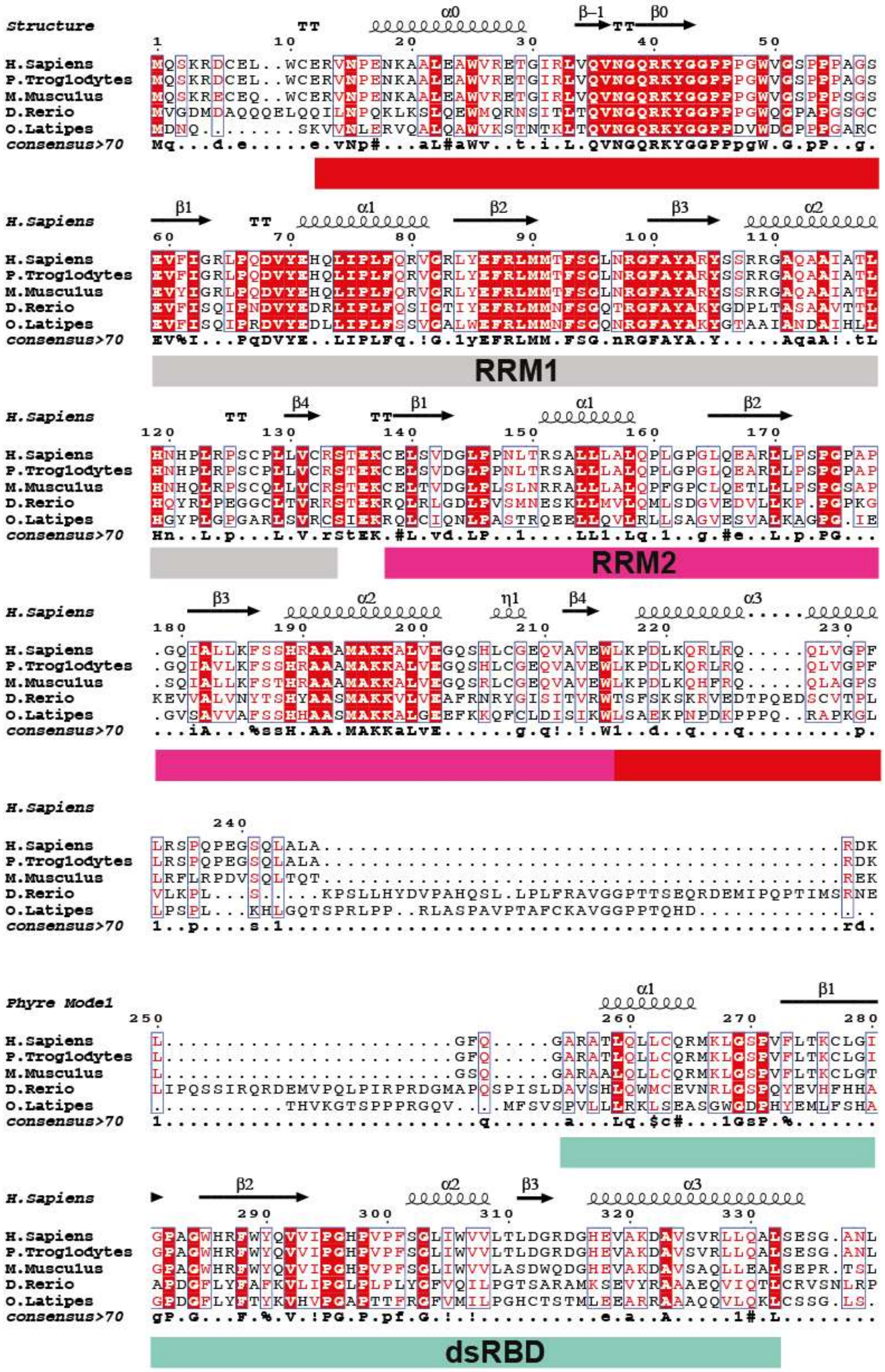

Structure
H.Sapiens
P.Troglodytes
M.Musculus
D.Rerio
O.Latipes
consensus>70
RRM1
RRM2
Phyre Model
dsRBD

D

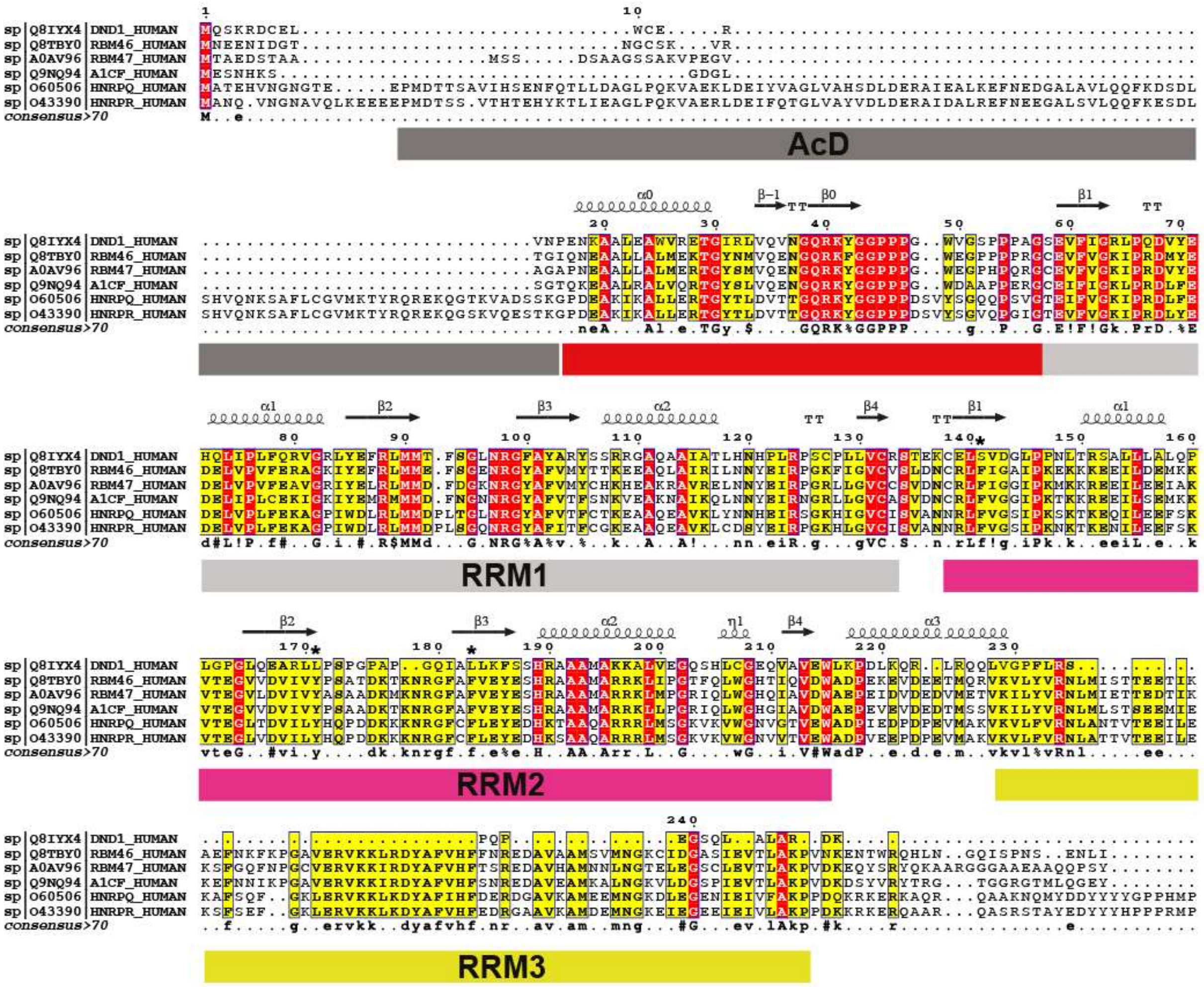

E

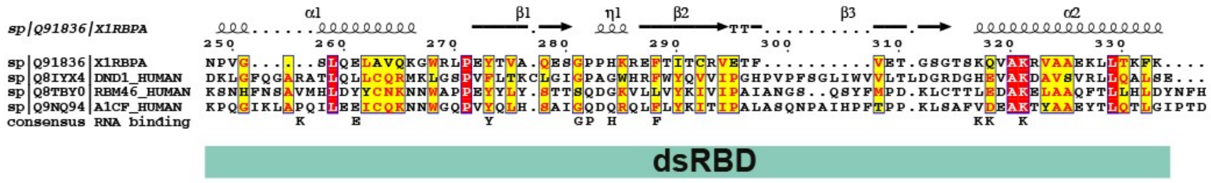

Supplementary Figure 1. Related to Figure 1.

A) Domain structures of the hnRNPR-like family of proteins that share sequence similarity to DND1 in its tandem RRMs, including the N-terminal extension. hnRNPQ and hnRNPR share a conserved N-terminal Acidic Domain (AcD) and C-terminal RGG repeats (Supplementary Fig. 1C). DND1, RBM46 and ACF share a dsRBD with low sequence conservation. Unique to DND1 is the lack of a third RRM, highly conserved in the other family members and lack of a canonical RNP RNA binding surface on RRM2. See Supplementary Fig. 1C for sequence alignments.

B) Schematic view of a canonical RRM adapted from PDBID 2UP1 [1]. The canonical RRM fold is βαββαβ folded into a four-stranded beta-sheet packed on top of two alpha-helices. The canonical RNA binding is of single-stranded RNA 5' to 3' over the beta-sheet starting at β4 towards β2. The central two beta-strands contain two conserved sequences RNP1 on β3 [R/K]-G-[F/Y]-[G/A]-[F/Y]-[I/L/V]-X-[F/Y] and RNP2 on β1 [I/L/V]-[F/Y]-[I/L/V]-X-N-L with three exposed aromatic sidechains (in green), two of which accommodate two bases by stacking interactions. In DND1 RRM1 these are F61 and Y102. A third aromatic sidechain (F100 in DND1 RRM1) usually stacks between the two ribose-moieties of these two nucleotides and an exposed long positively charged sidechain (in red) stabilizes the phosphate backbone between them. In DND1 RRM1 this is residue R98 that was mutated in the RIP assay (Fig. 1B, Supplementary Fig. 2).

C) Sequence alignment of DND1 from human (Uniprot Q8IYX4, RefSeq NP919225.1), chimp (Uniprot H2QRM7, RefSeq XP_517978.2), mouse (Uniprot Q6VY05, RefSeq NP_775559.2), zebrafish (Uniprot Q7T1H5, RefSeq NP_997960.1) and medaka (Uniprot C7DQU6, RefSeq NP_001157988.1), prepared with the programs T-Coffee [2] and ESPript 3.0 [3]. Phylogenetic analysis reveals a high level of conservation between species within the RNA binding domains RRM1, RRM2 and dsRBD but also the N-terminal extension of RRM1 (α0, β-1 & β0). Residues important for the interaction or discussed in the text are numbered according to the human protein sequence. Above the sequence alignment are the secondary structure elements based on either the tandem RRM12 structure presented in this paper or a model of the dsRBD calculated by the program PHYRE2 [4] based on PDB entry 2L2N. Beta-turns are annotated by 'TT'. Below is a consensus sequence using the following criteria: **u**ppercase is identity, **l**owercase is consensus level > 0.7, **!** is any of I/V, **$** is any of L/M, **%** is any of F/Y, **#** is any of N/D/Q/E/B/Z. Colors and boxes are displayed on the alignment according to similarity scores calculatedusing ESPript 3.0 [3]: Red box, white character = Strict identity, Red character = Similarity higher than 0.7, Blue frame = Similarity of stretch of residues higher than 0.7.

D) Sequence alignment of the N-terminal parts of the human hnRNPR-like family of RNA-binding proteins DND1, RBM46, RBM47, ACF, hnRNPQ and hnRNPR. Uniprot identifiers are indicated in front of the protein names. Alignment was prepared as described in Supplementary Fig. 1C. Non-canonical RNP residues in DND1 are indicated with an asterix above the sequence.

E) Sequence alignment of the dsRBDs of the human hnRNPR-like family of RNA-binding proteins DND1, RBM46 and ACF with the canonical second dsRBD of the protein XlRBPA with its secondary structure elements displayed above the sequence as found in PDBID 1DI2 [5]. Canonical dsRBD RNA binding residues are indicated below the sequence. Numbering according to DND1.

Supplementary Figure 2

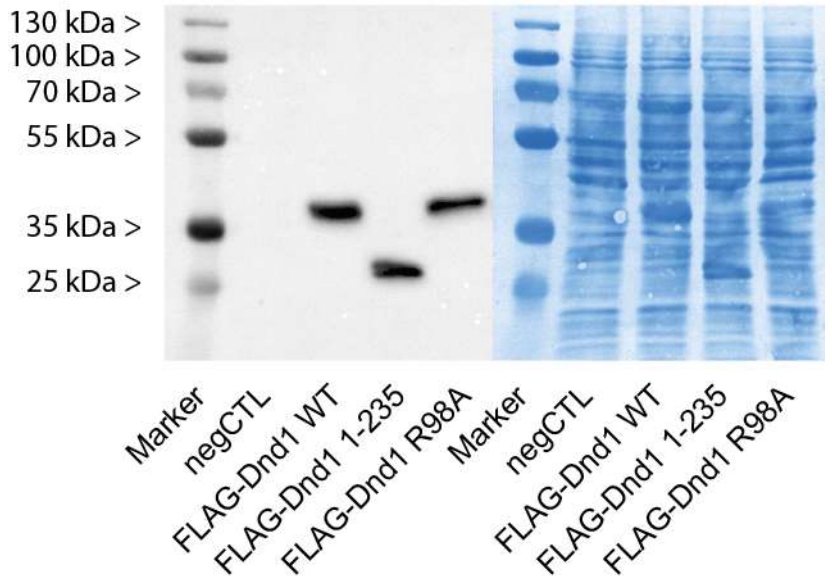

Supplementary Figure 2. Related to Figure 1B. Expression in HEK293T cell culture. Immunostaining with anti-FLAG antibody shows that DND1 and all mutants are well expressed in HEK293T cell culture. Western blot on the left, Coomassie staining of the same gel on the right. Source data are provided as a Source Data file. Western blots and Coomassie staining were performed on 7 independent transfections: twice to check protein stability and 5 times in 5 independent RIP experiments.

Supplementary Figure 3

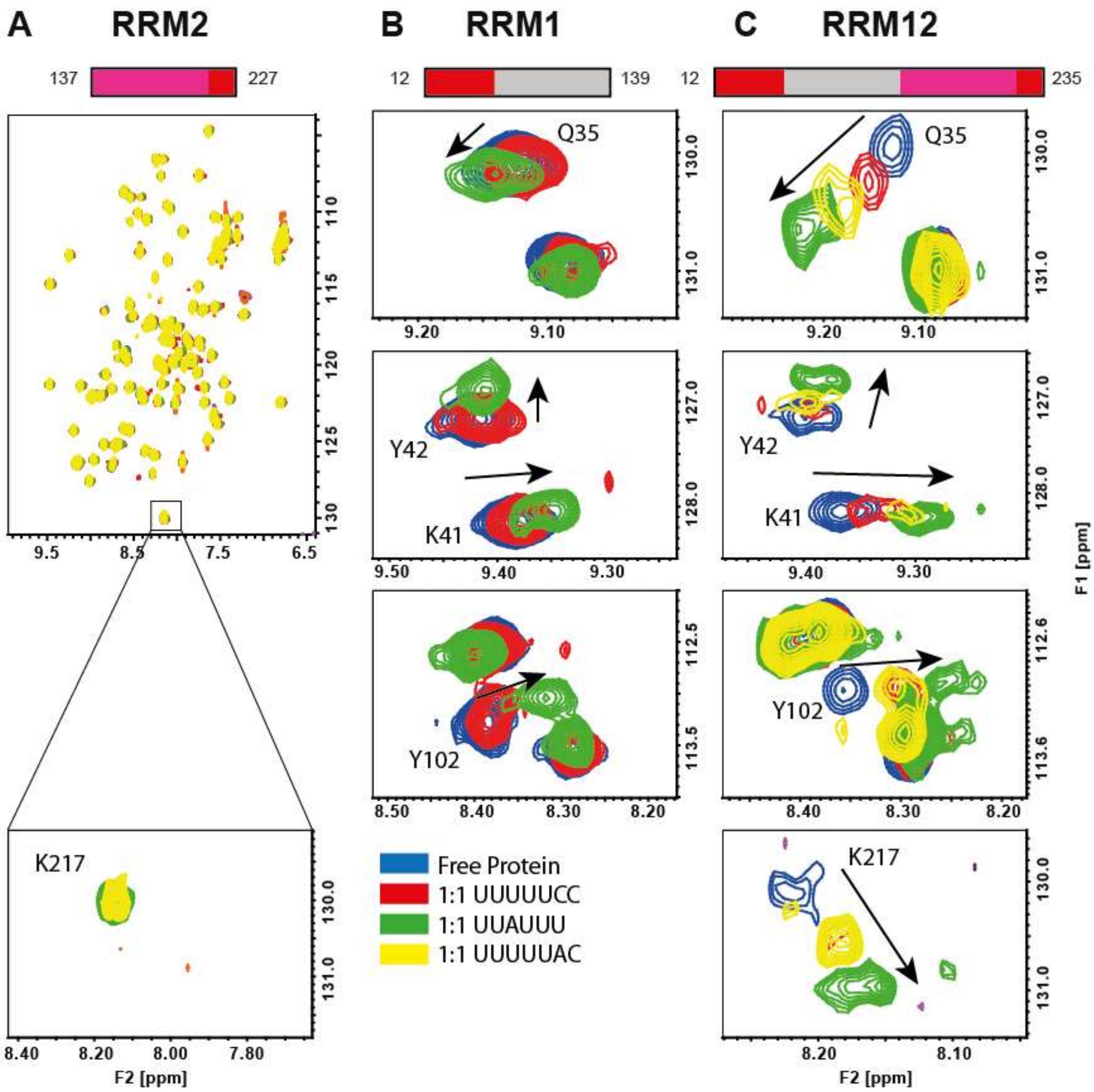

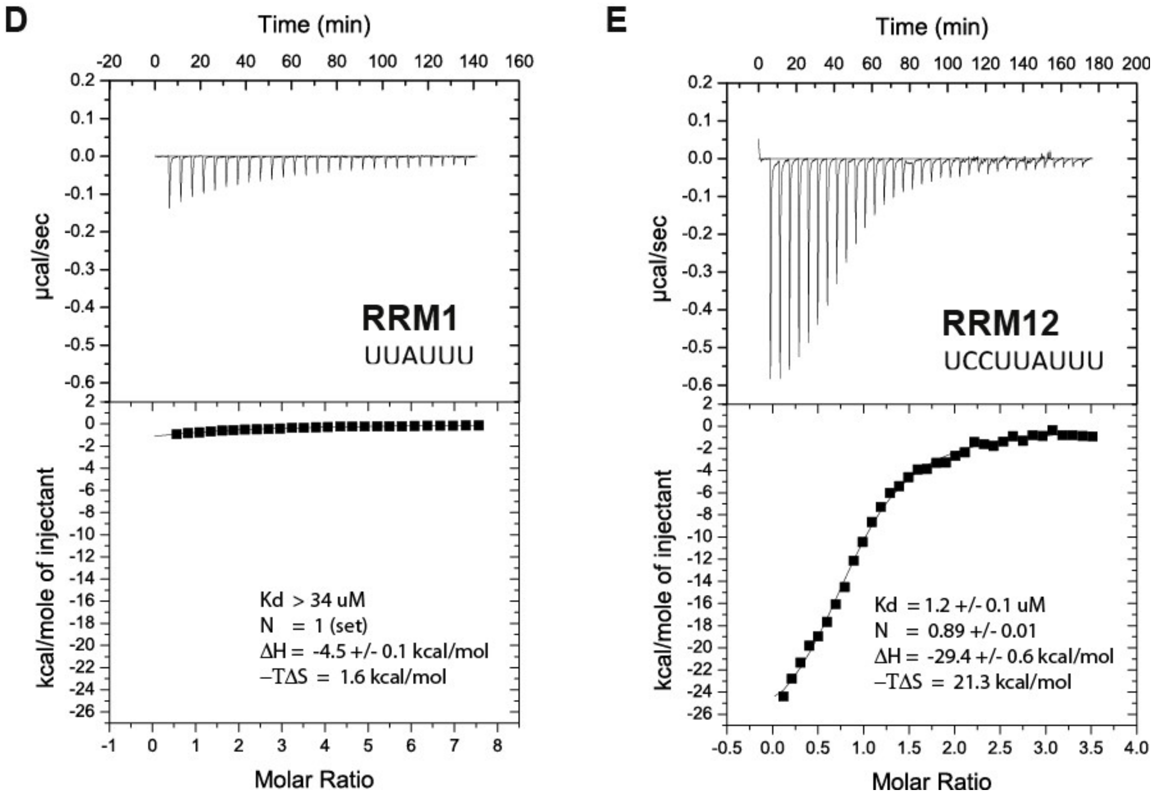

Supplementary Figure 3. Related to Figure 2. Finding the right protein construct and high affinity target for DND1-RNA structure determination using NMR titrations and ITC.

A/B/C) Overlay of free and RNA-bound ^{1}H-^{15}N-HSQC spectra of DND1

A) RRM2 includes a conserved C-terminal extension (137-227), full spectrum (top) and magnification of the individual cross-peak of the representative backbone amide of residue K217 in RRM2 alpha-helix 2

B) RRM1 includes a conserved N-terminal extension (12-139), magnification of residues Q35, K41 and Y42 in the N-terminal extension and Y102 in RNP1.

C) RRM12 is the tandem RRM including both extensions (12-235), magnification of Q35, K41, Y42 and K217. The free protein spectra are shown in blue, spectra of a 1:1 molar equivalent complex with the oligos UUUUUCC, UUAUUU and UUUUUAC in red, green and yellow respectively. RRM2 alone does not bind any of the oligos. The arrows show increasing chemical shift perturbations upon introduction of an adenine into the oligo. Largest perturbations, indicative of higher affinity binding, are seen upon addition of an RNA oligo containing a central adenosine to the tandem RRM construct. Although the oligo UUAUUU including a central adenine can bind RRM1 alone, it shifts the signals originating from the bound residues to much lesser extent than when bound to the tandem RRM12, reflecting an increase in binding affinity upon addition of RRM2 as is confirmed by ITC in Figure 2 in the main text. A U-rich oligo not containing adenines at all (UUUUUCC) is not bound by RRM1 alone. This oligo, as well as an

oligo with a peripheral adenine (UUUUUAC), is bound by the tandem RRM12 construct, although shifting the RNA bound residues to a lesser extent reflecting a weaker affinity. These titrations show that the affinity of DND1's tandem RRMs is highest to UUAUUU > UUUUUAC > UUUUUCC. NMR conditions: protein concentrations between 0.1 and 0.2mM, 298K, 750MHz. NMR buffer: 20mM MES pH 6.6, 100 mM NaCl, except for the RRM2 spectra recorded at 600MHz, in 25mM K_2HPO_4 / KH_2PO_4 buffer, 25mM NaCl, 2mM DTT pH6.3 at 303K.

D/E) Additional ITC binding experiments of RRM1 (D) and the tandem RRM12 (E) to AU-rich RNA show that when both domains are present, the affinity to AU-rich RNA is greatly increased compared to RRM1 alone. A titration with a longer AU-rich RNA (D) shows that an extension of the RNA at the 5' end around the central adenosine as was present in U-rich region 1 (URR1) targeted by DND1 in the p27/CDKN1B 3'UTR described by Kedde et al. [6] does not increase affinity for RRM12 binding (see also Supplementary Table 2).

Supplementary Figure 4

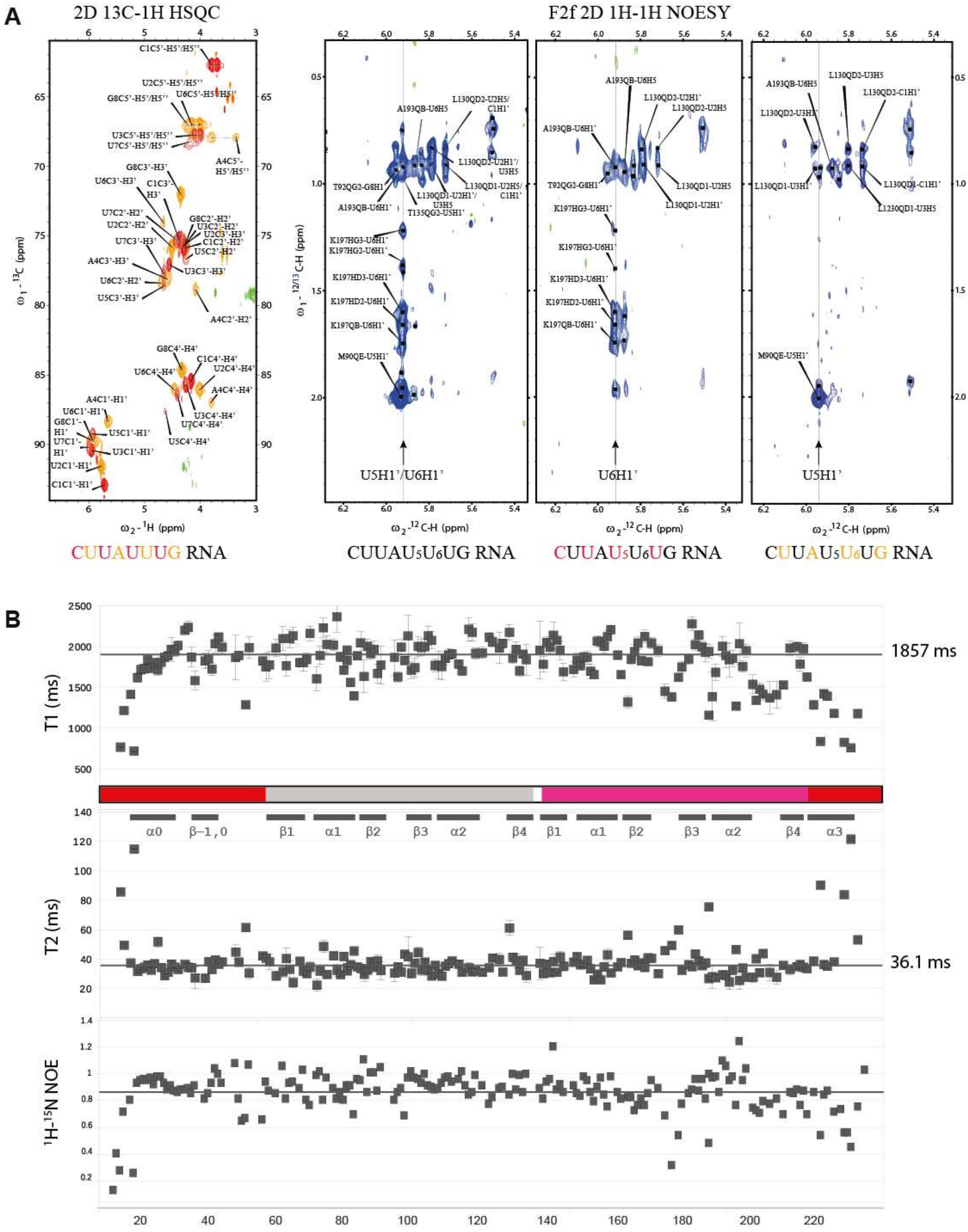

Supplementary Figure 4. Related to Figure 3. NMR data DND1 RRM12-CUUAUUUG complex

A) Selective ^{13}C-ribose labeling was essential for solving the structure of the DND1 tandem RRM – CUUAUUUG complex. Solid phase synthesis using ribose-^{13}C labeled phosphoramidites facilitated the synthesis of short RNA oligos with alternating ribose moieties labeled with the NMR active nucleus ^{13}C. Overlay of 2D ^{1}H-^{13}C-HSQC spectra of CUUAUUUG RNA ^{13}C ribose-labeled in either the 1st, 3rd, 5th and 7th (in red) or 2nd, 4th, 6th and 8th (in yellow) nucleotide position, in complex with the unlabeled tandem RRMs of DND1. Assignment of the heavily overlapped ribose protons is facilitated by resolution in the ^{13}C dimension and reduction of assignment possibilities due to the selective labeling B) Excerpts of 2D NOESY spectra of complexes between the DND1 ^{13}C^{15}N-labeled tandem RRMs and either unlabeled or selectively ^{13}C-ribose labeled CUUAUUUG RNA, ^{13}C filtered in the F2 dimension (F2f). Cross-peaks represent short distances (under 5 Ångstrom) between protons attached to unlabeled carbons only in the F2 dimension (in this case RNA base and unlabeled ribose) and protons attached to both ^{13}C-labeled carbons (all protein and RNA labeled ribose) and unlabeled carbons (RNA base and unlabeled ribose) in F1. The arrow indicates the frequency at which both the U_5 and U_6 H1' protons resonate. By selectively labeling the U_5 ribose the cross-peaks to its H1' can be suppressed with a ^{13}C-filter thus revealing NOEs specific to the U_6 ribose whereas a sample incorporating 13C-labeled U_6 ribose allows detection of NOEs to U_5, making unambiguous assignment of the short distances between the protein sidechains and the U_5 and U_6 H1' protons possible.

B) Backbone dynamics of the DND1 tandem RRM-CUUAUUUG complex. ^{15}N-T1 and ^{15}N-T2 relaxation rates and heteronuclear {^{1}H}-^{15}N NOEs (HetNOE) were measured by standard techniques described in the Method details and are plotted against the protein sequence where RRM1 is shown in grey, RRM2 in pink and the N- and C-terminal extensions are in red. Secondary structure elements are indicated below the sequence. Data are presented as mean +/- SD of the relaxation rates. SD was estimated by Monte-Carlo error analysis (21 simulations), using the errors in the peak intensities with automated estimation of the spectral noise (non-linear least squares fit to a mono-exponential decay using modelXY in NMRPipe [7]). Similar T1/T2 ratios for both RRMs including the N-terminal extension suggest isotropic motion, except for the unfolded N- terminal tail, the C-terminal α3-helix and several loop residues. The average values of T2 and T1 of all residues with these exceptions indicated by solid lines are consistent with a globular protein of approximately 30 kDa. This is consistent with the size of a monomeric DND1 tandem RRM-CUUAUUUG complex determined by extrapolating from the correlation of τ_c and MW recorded at the same temperature (298K) and a similar field strength [8]. A rotational correlation time (τ_c) of 18ns was estimated using the formula

$$\tau_c \approx \left(\sqrt{\frac{6T_1}{T_2} - 7}\right) / 4\pi\nu_N$$

derived from Eq. 8 in Kay et al. [9] which considers only the contributions of $J(0)$ and $J(\omega_N)$ spectral density terms to T1 and T2 relaxation, neglecting higher frequency terms, and where ν_N is the ^{15}N resonance frequency (76,01 MHz). Source data are provided as a Source Data file.

Supplementary Figure 5

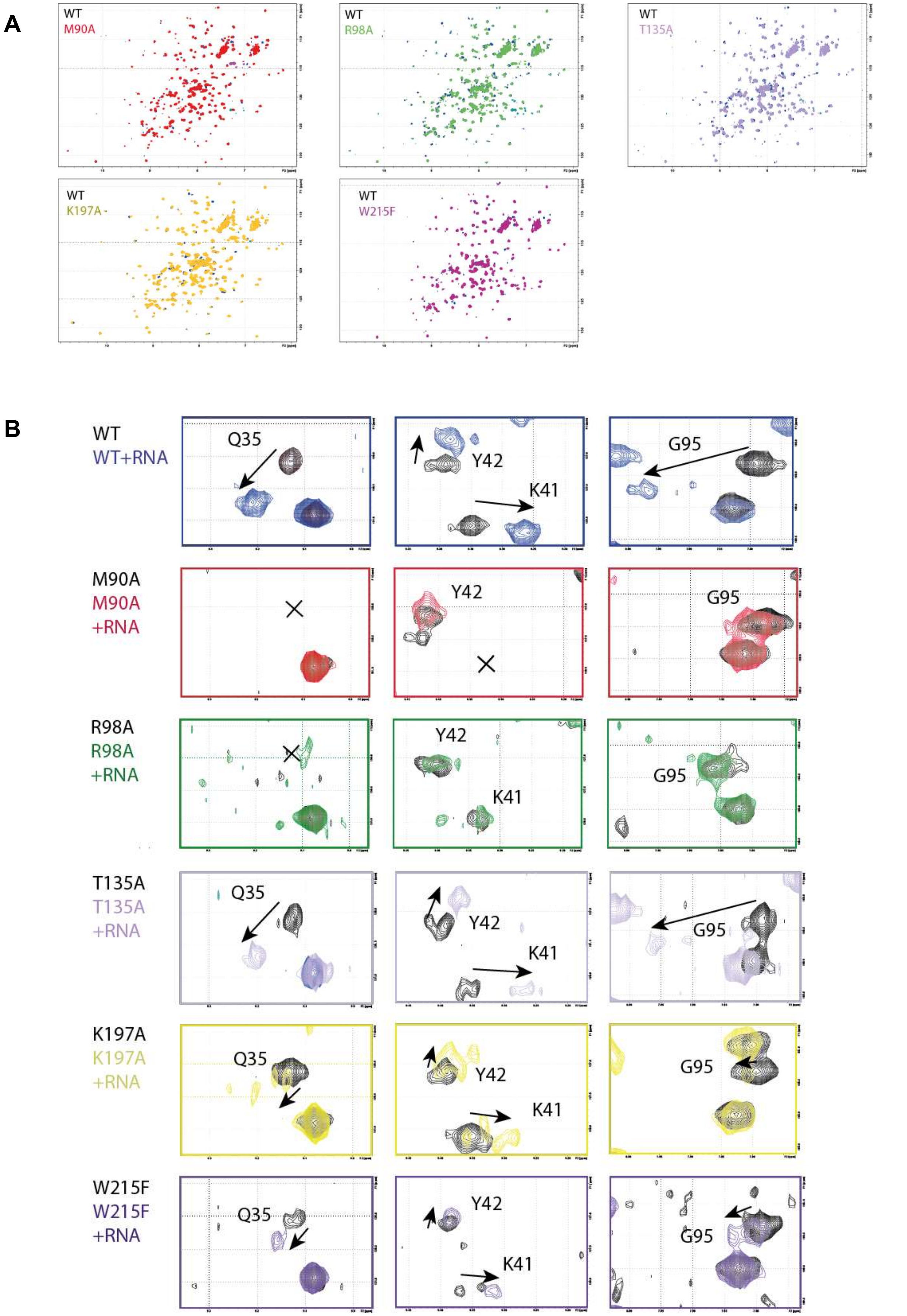

C

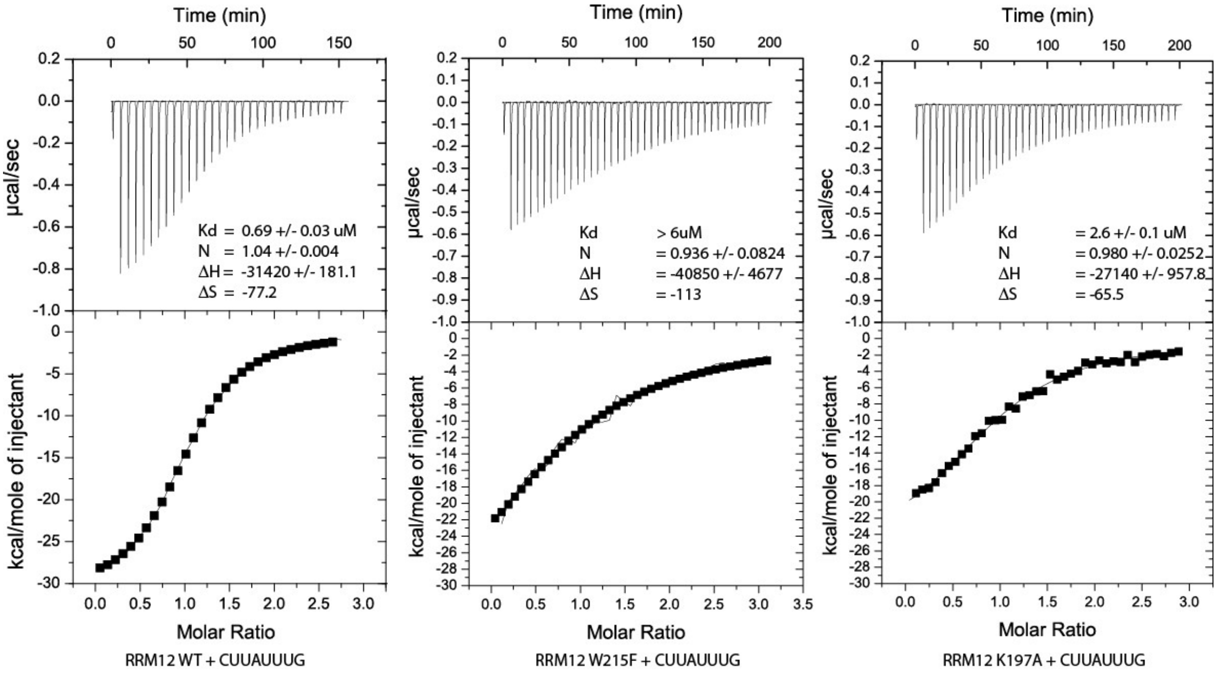

Supplementary Figure 5. Related to Figure 4. Mutational analysis of DND1 tandem RRM binding to CUUAUUUG RNA.

A) ^{1}H-^{15}N HSQC spectra of the WT tandem RRMs of DND1 are overlaid individually with the mutants M90A, R98A, T135A, K197A and W215F and show that all these mutants are correctly folded

B) Disappearance or diminishing of chemical shift perturbations upon titration of CUUAUUUG RNA to several mutants compared to the WT tandem RRMs is observed for several reporter residues.

C) ITC measurement of DND1's tandem RRMs domain titrated with CUUAUUUG RNA. WT: N = 1.04 +/- 0.004; K_D = 0.69 +/- 0.03 uM; ΔH = -31.4 +/- 0.2 kcal/mol; $-T\Delta S$ = 23.0 kcal/mol; W215F: N=0.919 +/- 0.172; K_D = > 10uM; ΔH = -68 +/- 15 kcal/mol; $-T\Delta S$ = 61.4 kcal/mol; K197A: N=0.98 +/- 0.03; K_D = 2.6 +/- 0.1 uM; ΔH = -27.1 +/- 1.0 kcal/mol; $-T\Delta S$ = 19.5 kcal/mol.

Supplementary Figure 6

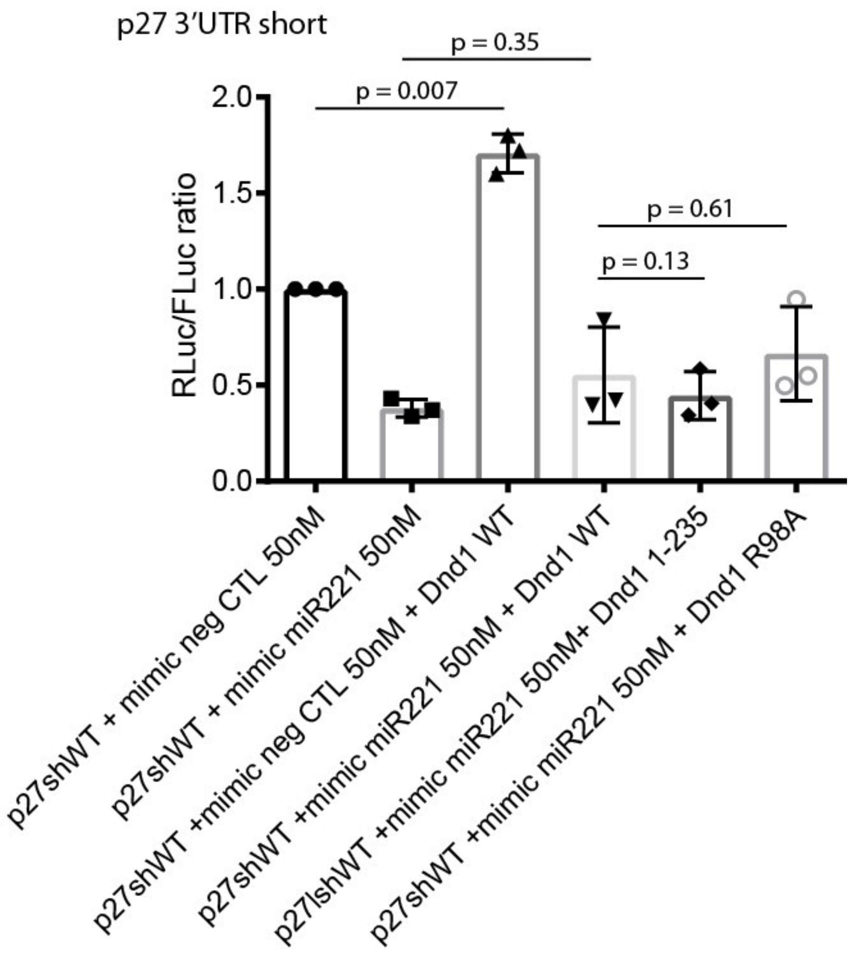

Supplementary Figure 6. Related to Figure 5. DND1 increases p27 3'UTR controlled reporter gene expression independent of the presence of a miR-mimic targeting this UTR or a negative control. A MiRIDIAN 221-3p miRNA mimic or negative control mimic was cotransfected into HEK293T with a short p27 UTR psiCHECK-2 luciferase construct and either WT or mutant FLAG-tagged DND1. Relative luciferase activity is the ratio between Renilla control and Firefly luciferases, adjusted to 1 for 100%. The results are represented as means and SD from three independent experiments. p-values obtained from two-tailed Welch's t-test. Source data are provided as a Source Data file.

Supplementary Figure 7

A) Proteins enriched in DND1 WT vs R98A RNA binding mutant interactome:

(DND1) ELAVL1, HNRNPL, PHAX, DHX30, PABPC1, SYNCRIP, MATR3, MOV10, MEX3A, TNRC6B, DHX9, HNRNPA2B1, LARP1, ILF3, SART3, HNRNPC, FUBP3

GO Biological Process 2018 ranked by EnrichR [10] combined score:

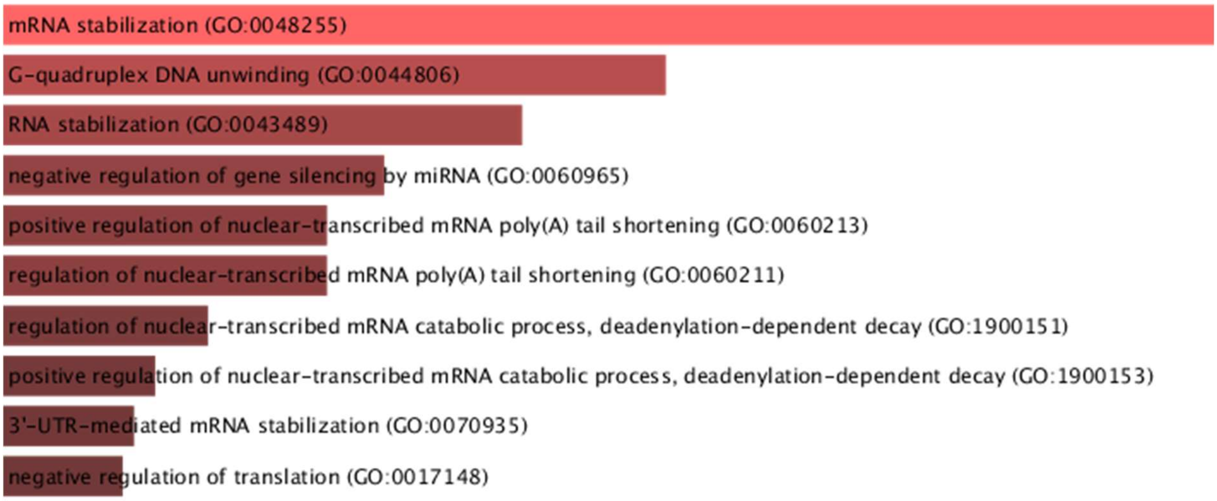

Index	Name	P-value	Adjusted p-value	Odds Ratio	Combined score
1	mRNA stabilization (GO:0048255)	2.282e-13	1.164e-9	202.02	5880.52
2	G-quadruplex DNA unwinding (GO:0044806)	0.00001602	0.006289	317.46	3505.25
3	RNA stabilization (GO:0043489)	4.129e-7	0.0002341	196.08	2882.36
4	negative regulation of gene silencing by miRNA (GO:0060965)	0.00003428	0.01093	222.22	2284.67
5	positive regulation of nuclear-transcribed mRNA poly(A) tail shortening (GO:0060213)	0.00004187	0.01257	202.02	2036.54
6	regulation of nuclear-transcribed mRNA poly(A) tail shortening (GO:0060211)	0.00004187	0.01187	202.02	2036.54
7	regulation of nuclear-transcribed mRNA catabolic process, deadenylation-dependent decay (GO:1900151)	0.00006917	0.01681	158.73	1520.46
8	positive regulation of nuclear-transcribed mRNA catabolic process, deadenylation-dependent decay (GO:1900153)	0.00009112	0.02114	138.89	1292.13
9	3'-UTR-mediated mRNA stabilization (GO:0070935)	0.0001032	0.02290	130.72	1199.83
10	negative regulation of translation (GO:0017148)	1.204e-8	0.00001024	63.13	1151.22

GO Cellular Component 2018 ranked by EnrichR [10] combined score:

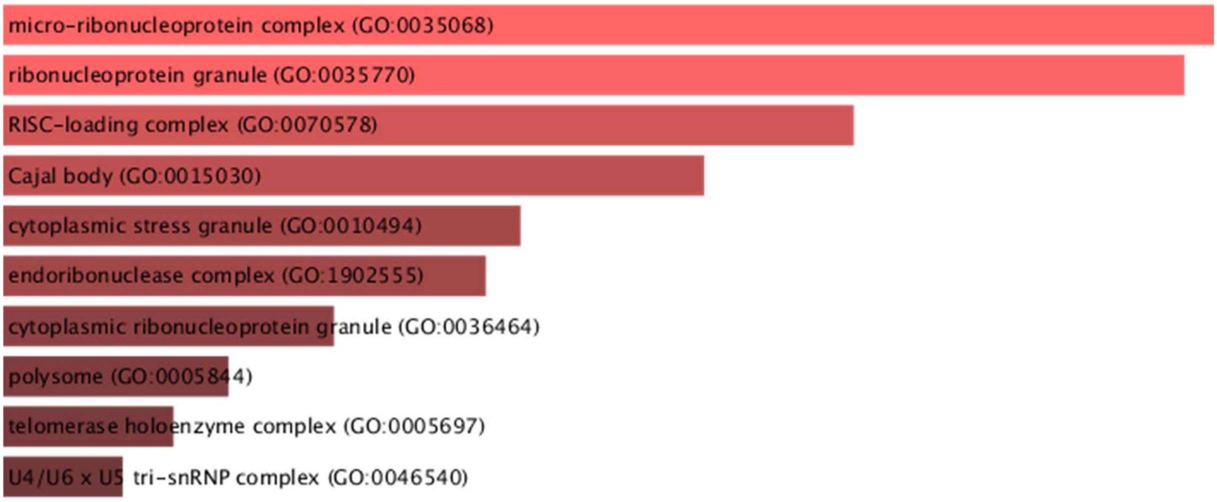

Index	Name	P-value	Adjusted p-value	Odds Ratio	Combined score
1	micro-ribonucleoprotein complex (GO:0035068)	0.006284	0.4004	158.73	804.73
2	ribonucleoprotein granule (GO:0035770)	6.958e-7	0.0003103	55.56	787.68
3	RISC-loading complex (GO:0070578)	0.008072	0.4500	123.46	594.98
4	Cajal body (GO:0015030)	0.0004219	0.06272	65.36	507.89
5	cytoplasmic stress granule (GO:0010494)	0.0006144	0.06851	54.20	400.81
6	endoribonuclease complex (GO:1902555)	0.01164	0.5768	85.47	380.62
7	cytoplasmic ribonucleoprotein granule (GO:0036464)	0.00001405	0.003133	26.14	292.11
8	polysome (GO:0005844)	0.001446	0.1290	35.27	230.64
9	telomerase holoenzyme complex (GO:0005697)	0.01962	0.7957	50.51	198.54
10	U4/U6 x U5 tri-snRNP complex (GO:0046540)	0.02227	0.7641	44.44	169.09

B) Proteins enriched in DND1 WT vs 1-235 dsRBD truncation mutant interactome:

(DND1) TRAP1, TNRC6B, HSPA1A, MATR3, MTHFD1, MEX3A, HSPA6, DDX17, HNRNPC

GO Molecular Function 2018 ranked by EnrichR [10] p-values:

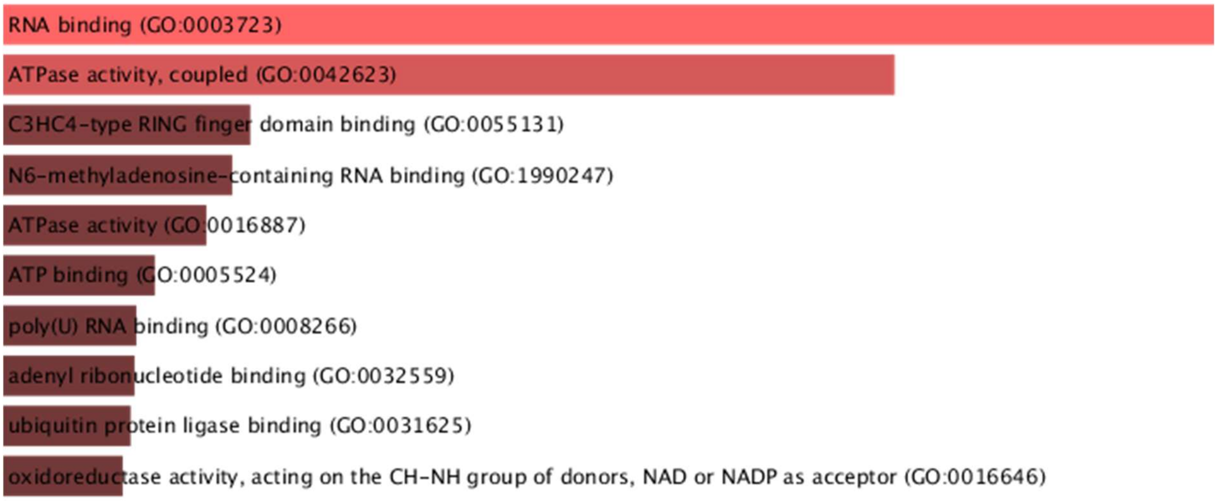

Index	Name	P-value	Adjusted p-value	Odds Ratio	Combined score
1	C3HC4-type RING finger domain binding (GO:0055131)	0.002997	1.000	333.33	1936.76
2	N6-methyladenosine-containing RNA binding (GO:1990247)	0.003495	1.000	285.71	1616.10
3	ATPase activity, coupled (GO:0042623)	0.00001178	0.006779	63.83	724.41
4	poly(U) RNA binding (GO:0008266)	0.007973	1.000	125.00	603.96
5	oxidoreductase activity, acting on the CH-NH group of donors, NAD or NADP as acceptor (GO:0016646)	0.008966	1.000	111.11	523.82
6	poly-pyrimidine tract binding (GO:0008187)	0.009957	0.9551	100.00	460.95
7	AU-rich element binding (GO:0017091)	0.01045	0.9255	95.24	434.37
8	telomerase RNA binding (GO:0070034)	0.01095	0.9001	90.91	410.42
9	disordered domain specific binding (GO:0097718)	0.01144	0.8781	86.96	388.73
10	miRNA binding (GO:0035198)	0.01194	0.8588	83.33	369.00

Supplementary Figure 7. Related to Figure 6. EnrichR [10] Gene Ontology analysis. Proteins differentially present (multiple two-sided *t*-tests, FDR 10%) in WT vs mutant A) R98A; B) 1-235 DND1 HEK293T interactomes are listed, followed by GO-enrichment analysis, correcting for multiple comparisons as described in Chen et al. [10].

Supplementary Figure 8

A

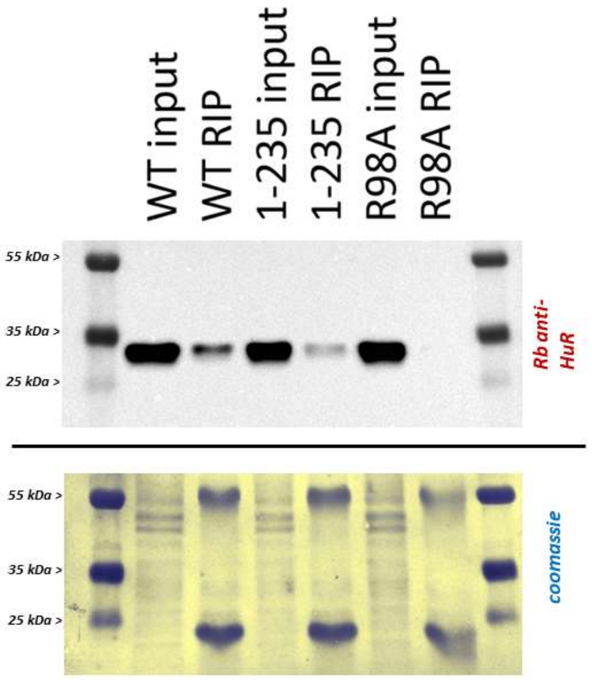

B

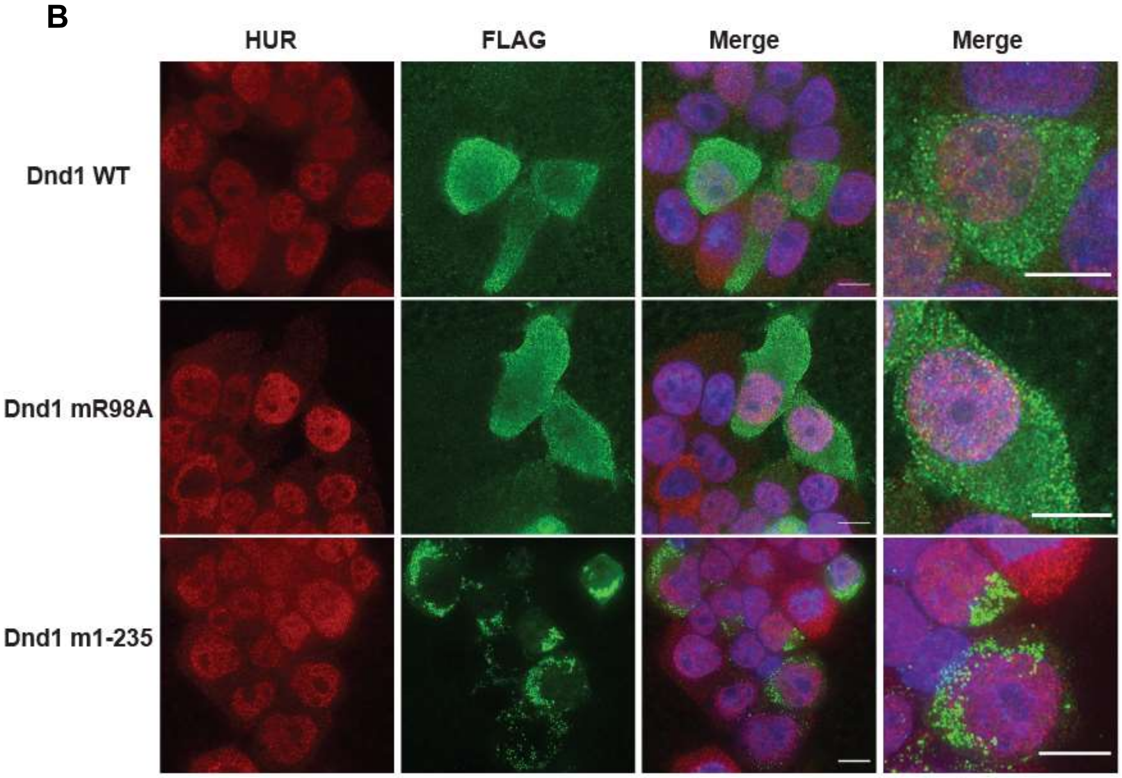

C

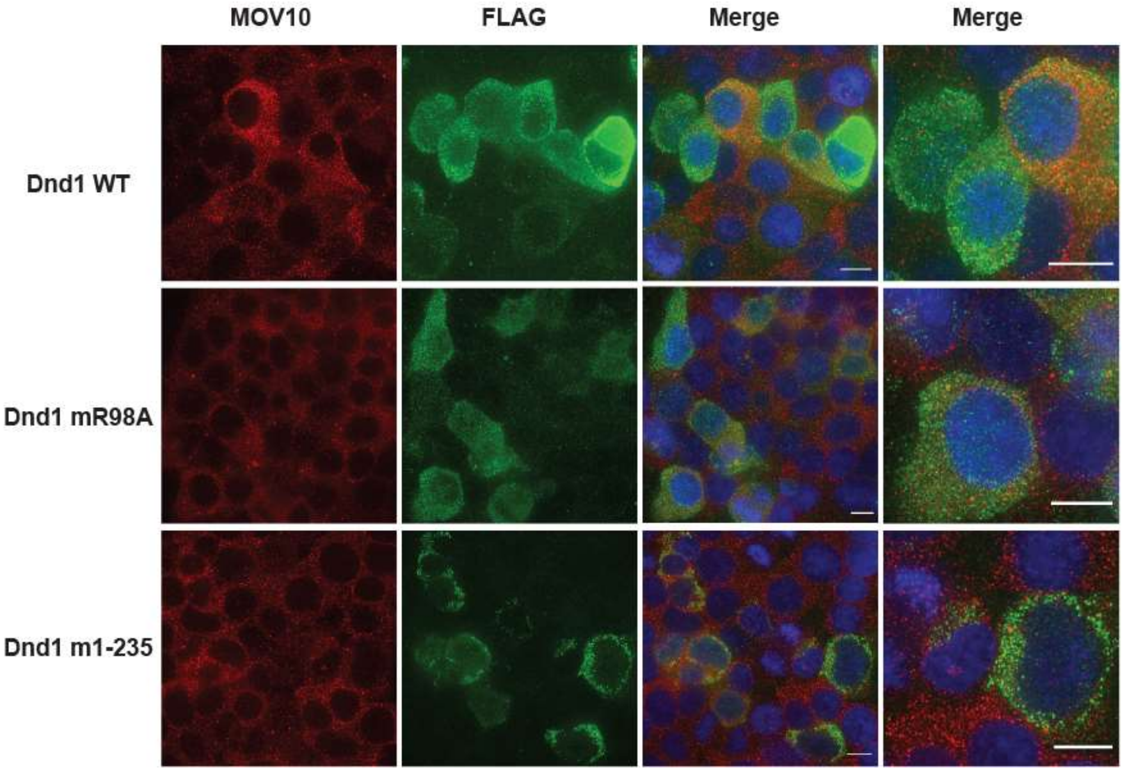

Supplementary Figure 8. Related to Figure 6.

A) Western blot of ELAVL1/HuR from our WT, R98A and 1-235 DND1 transfected HEK293T pulldowns shows enrichment in WT and 1-235 dsRBD pulldowns only. Representative blot form three independent transfections followed by pulldowns. Source data are provided as a Source Data file.

B) Indirect immunofluorescence (IF) experiments in HEK293T cells transiently transfected with plasmids encoding the indicated FLAG tagged Dnd1 proteins. HUR is shown in red, FLAG in green and DAPI stained nuclei in blue. Scale bar 10μm C) MOV10 is shown in red, FLAG in green and DAPI stained nuclei in blue. Scale bar 10μm. Representative images from three independent transfections.

C) Indirect immunofluorescence (IF) experiments in HEK293T cells transiently transfected with plasmids encoding the indicated FLAG tagged Dnd1 proteins MOV10 is shown in red, FLAG in green and DAPI stained nuclei in blue. Scale bar 10μm. Representative images from three independent transfections.

Supplementary Table 1. Related to Figure 1, Supplementary Figure 2 and Methods. Primers.

Primer name	Primer sequence
Primers for qPCR	
SMN_f	TGAGCTGTGAGAAGGGTGTT
SMN_r	ACAGCCATGTCCACCAGTTAG
PMAIP_f	CTG GGC TAT ATA CAG TCC TCA AA
PMAIP_r	AGT GAC TAC AAC CTA CAT TAA GAC A
SRSF2_f	AGA TCT AAA TGG TAA TCT GAA CCC A
SRSF2_r	CAA CAC GCC ACC TTA ACA GC
SLC25A6_f	CAG CCG ATT CCG TGT CTT GA
SLC25A6_r	GGG CTA GCG CTG AAG TAC AA
Primers for protein constructs	
hsDnd1_S1	CGG GAT CCA TGC AGT CCA AGC GGG AT
hsDnd1_As353	CGG GAA TTC CTA TCA TTA CTG TTT AAC CAT GGT ACC TGC CTC
hsDnd1_S12_BbsI_NcoI	CTA GAA GAC ACC ATG GAG AGG GTG AAT CCA GAG AAC AAG
hsDnd1_S136_BbsI_NcoI	CTA GAA GAC ACC ATG GAG AAG TGT GAG CTG AGC GTT GAC
hsDnd1_As139_Acc65I	GCT AGG TAC CTT ACT CAC ACT TCT CGG TGC TG
hsDnd1_As227_Acc65I	GCT AGG TAC CTT ACT GCT GGC GAA GTC GCT GCT TC
hsDnd1_As235_Acc65I	GCT AGG TAC CTT AGG ACC GCA AGA AGG GAC CCA C
Primers for protein mutations	
Mut_Dnd1_M90A_f	CGA GTT CCG CCT GGC GAT GAC CTT CAG
Mut_Dnd1_M90A_r	CTG AAG GTC ATC GCC AGG CGG AAC TCG
Mut_Dnd1_R98A_f	CAG CGG CCT GAA CGC CGG CTT CGC CTA TG
Mut_Dnd1_R98A_r	CAT AGG CGA AGC CGG CGT TCA GGC CGC TG
Mut_Dnd1_T135A_f	GTG TGC CGC AGC GCC GAG AAG TGT G
Mut_Dnd1_T135A_r	CAC ACT TCT CGG CGC TGC GGC ACA C
Mut_Dnd1_K197A_f	GCC ATG GCC AAA GCG GCC CTG GTG G
Mut_Dnd1_K197A_r	CCA CCA GGG CCG CTT TGG CCA TGG C
Mut_Dnd1_W215F_f	GTG GCT GTG GAG TTC CTC AAG CCA GAC C
Mut_Dnd1_W215F_r	GGT CTG GCT TGA GGA ACT CCA CAG CCA C
Primers for luciferase assay reporter constructs	
p27_sh3UTR_f_XhoI	GCT ACT CGA GGC CTC TAA AAG CGT TGG ATG
p27_sh3UTR_r_NotI	GCT AGC GGC CGC GTG CTA CAT AAA AGG TAA AAA CTA TAT ACA CAG
TERT_3UTR_f_XhoI	AAA ACT CGA GCG AGA GCA GAC ACC
TERT_3UTR_r_NotI	AAA AGC GGC CGC TTT TAC TCC CAC
TERT_mut_UAUUU_f	GCC CGG CTT TAT TTC CAC TCC CCA CAT AGG AAT AGT CCA TC
TERT_mut_UAUUU_r	GGG GAG TGG AAA TAA AGC CGG GCT CCT GGT GAG G
TERT_mut_UAUUU_ UUUU_UAUUU_f	GCC CGG CTT TAT TTT TTT TAT TTC CAC TCC CCA CAT AGG AAT AGT CCA TC
TERT_mut_UAUUU_ UUUU_UAUUU_r	GGG GAG TGG AAA TAA AAA AAA TAA AGC CGG GCT CCT GGT GAG G

Supplementary Table 2. Related to Figure 2 and Supplementary Fig. 3. Summary of *in vitro* protein-RNA interaction studies of the Dnd1 individual and tandem RRM domains with different RNA oligos.

oligo	RRM2	RRM1		RRM12	
	NMR	NMR	ITC (μM)	NMR	ITC (μM)
UUUUUCC	-	-		+	> 100
UUUUUAC	-	-		++	> 35
UUAUUU	-	+	> 35	+++	
CUUAUUUG	-	+	> 54	+++	0.68
UCCUUAUUU					1.2

'-' means lack of binding in an NMR titration. Increasing numbers of '+' indicate binding with increasing affinity as indicated by increasing chemical shift perturbations of backbone amide proton NMR signals in a ^{1}H-^{15}N HSQC spectrum (see Supplementary Fig. 3). NMR buffer: 20mM MES pH 6.6, 100 mM NaCl. ITC buffer: 100 mM $KHPO_4$/ KH_2PO_4 pH 6.6, 1mM 2-mercaptoethanol.

Supplementary Table 3: NMR statistics

Dnd1(12-235):CUUAUUUG		
NMR Restraints		
Distance Restraints		5200
Protein	intramolecular	4947
	intraresidual	942
	sequential (|i-j|=1)	1313
	medium range (1<|i-j|<5	1190
	long range (|i-j|>=5	1423
	hydrogen bonds[a]	79
RNA	intramolecular	150
	intraresidual	116
	sequential (|i-j|=1)	34
	medium range (1<|i-j|<5)	0
	long range (|i-j|>=5)	0
	hydrogen bonds	0
Complex	intermolecular	103
	long range (|i-j|>=5	103
	hydrogen bonds	0
Torsion Angles[b]		
Protein	backbone	176
RNA	sugar pucker (DELTA)	8
RDCs		
Protein	amide NH	127
Energy Statistics[c]		
Average distance constraint violations		
0.3-0.4 Å		10.3 +/- 2.5
>0.4 Å		5.5 +/- 2.0
Maximal (A)		0.66 +/- 0.14
Average angle constraint violations		
>5 degree		3.0 +/- 1.2
Maximal (degree)		9.0 +/- 4.5
Average RDC violations		
>5 Hz		5.2 +/- 1.2
Maximal (Hz)		6.1 +/- 0.9
RDC correlation coefficient		0.96 +/- 0.01
Q = rms(Dcalc-Dobs)/rms(Dobs) (%)		30.7 +/- 2.2
Q normalized by tensor		17.9 +/- 1.5
Mean AMBER Violation Energy		
Constraint (kcal mol^{-1})		297.5 +/- 21.9
Distance (kcal mol^{-1})		168.9 +/- 18.5
Torsion (kcal mol^{-1})		10.7 +/- 2.4
Alignment (kcal mol^{-1})		104.4 +/- 9.3
Mean AMBER Energy (kcal mol^{-1})		-7568.9 +/- 18.5
Mean Deviation from ideal covalent geometry		
Bond Length (A)		0.0041 +/- 0.0000
Bond Angle (degrees)		1.511 +/- 0.009
Ramachandran plot Statistics[c,d]		
Residues in most favoured regions (%)		82.1 +/- 1.9
Residues in additionally allowed regions (%)		15.3 +/- 2.1
Residues in generously allowed regions (%)		2.0 +/- 0.8
Residues in disallowed regions (%)		0.6 +/- 0.5
RMSD to mean structure		
Protein Dnd1 RRM1 16-133		
Backbone atoms		0.95 +/- 0.23
Heavy atoms		1.23 +/- 0.20
Protein Dnd1 RRM2 134-216		
Backbone atoms		0.57 +/- 0.12
Heavy atoms		0.92 +/- 0.12
Protein Dnd1 RRM12 16-216		
Backbone atoms		1.05 +/- 0.14
Heavy atoms		1.36 +/- 0.13
RNA UAUUU		
Backbone atoms		0.43 +/- 0.13
Heavy atoms		0.60 +/- 0.18
Complex RRM12 16-216;UAUUU		
Backbone atoms		1.04 +/- 0.14
Heavy atoms		1.26 +/- 0.13

RMSD, root-mean-square deviation
a Hydrogen bond constraints were identified from slow exchanging amide and imino protons in D_2O
b Torsion angle based on TALOS+ predictions; sugar puckers based on homonuclear TOCSY
c Dnd1 RRM12, 16-216 Chain ID: A (Sequence Range:12-235); RNA CUUAUUUG: 3-7, Chain ID: B (Sequence Range:1-8)
d Ramachandran plot, as defined by the program Procheck[11].

Supplementary Table 4. Related to Methods section. Reagents & resources table

REAGENT or RESOURCE	SOURCE	IDENTIFIER
Antibodies		
FLAG-M2-HRP	Sigma	A8592
HuR rabbit polyclonal antibody	ProteinTech	11910-1-AP
MOV10 rabbit polyclonal antibody	ProteinTech	10370-1-AP
Alexa fluor 488 donkey anti-mouse IgG	Life Technologies	A21202
Alexa fluor 546 donkey anti-rabbit IgG	Life Technologies	A10040
Bacterial and Virus Strains		
Top10 competent cells	This study	
BL21(DE3) competent cells	This study / Novagen	
Biological Samples		
DMEM	Sigma	D6429
FBS	Sigma	F7524
OptiMEM	GIBCO	31985047
Chemicals, Peptides, and Recombinant Proteins		
TEV protease	In-house produced	
Critical Commercial Assays		
Clarify ™ Western ECL substrate	BioRad	170-5061
Dual-Glow Luciferase Assay System	Promega	E2920
Deposited Data		
Chemical Shifts	BioMagResBank	34675
Structural Ensemble	Protein Data Bank	7Q4L
Experimental Models: Cell Lines		
HEK293T cell line	ATCC	CRL-3216™
Oligonucleotides		
CUUAUUUG RNA	Dharmacon	
selectively ribose-13C labeled RNA	This study	
Oligonucleotides for cloning & site-directed mutagenesis	Microsynth	Supplementary Table 1
miRIDIAN miR-221-3p miRNA mimic	Dharmacon	MIMAT0000278
Scrambled miRNA mimic	Dharmacon	CN-001000-01-05
Recombinant DNA		
pET-M11 vector	EMBL	
pET-His-TEV	EMBL	
psiCHECK2 vector	Promega	
pCMV-SPORT6-hsDnd1 source plasmid	Source BioScience	IRATp970F0747D
pcFLAG_DNA3.1	This study / Invitrogen	
Software and Algorithms		
NMR data acquisition and processing	Topspin (Bruker)	Version 2.1
NMR data processing and relaxation analysis	NMRPipe	
Spectral analysis	NMRFAM-Sparky	Version 1.1412
Structure refinement	AMBER 12	Ff12SB force field
Structure calculation	CYANA	Version 3.98
Peak picking, structure calculation	ATNOS-CANDID	Version 3.1
Statistical analyses	PRISM	Version 7.04
MS/MS sample protein identification	Mascot	Version 2.6.2
MS/MS peptide and protein validation	Scaffold	Version 4.8.9

Supplementary References

1. Ding J, Hayashi MK *et al.* Crystal structure of the two-RRM domain of hnRNP A1 (UP1) complexed with single-stranded telomeric DNA. *Genes Dev* **13**: 1102-1115 (1999).

2. Notredame C, Higgins DG, Heringa J. T-coffee: a novel method for fast and accurate multiple sequence alignment. *J Mol Biol* **302**: 205–217 (2000).

3. Robert X, Gouet P. Deciphering key features in protein structures with the new ENDscript server. *Nucleic Acids Res* **42**: W320–W324 (2014).

4. Kelley LA, Mezulis S, Yates CM, Wass MN, Sternberg MJE. The Phyre2 web portal for protein modeling, prediction and analysis. *Nat Protoc* **10**: 845–858 (2015).

5. Ryter JM, Schultz SC. 1998. Molecular basis of double-stranded RNA-protein interactions: structure of a dsRNA-binding domain complexed with dsRNA. *EMBO J* **17**: 7505–13 (1998).

6. Kedde, M. *et al.* RNA-binding protein Dnd1 inhibits microRNA access to target mRNA. *Cell* **131**, 1273–86 (2007).

7. Delaglio, F. *et al.* NMRPipe: A multidimensional spectral processing system based on UNIX pipes. *J. Biomol. NMR* **6**, 277–293 (1995).

8. Aramini JM, Tubbs JL, *et al.* Structural basis of O6-alkylguanine recognition by a bacterial alkyltransferase-like DNA repair protein. *J Biol Chem* **285:** 13736–41 (2010).

9. Kay LE, Torchia DA, Bax A. Backbone dynamics of proteins as studied by nitrogen-15 inverse detected heteronuclear NMR spectroscopy: application to staphylococcal nuclease. *Biochemistry* **28**: 8972–8979 (1989)

10. Chen EY, Tan CM, Kou Y, Duan Q, Wang Z, Meirelles GV, Clark NR, Ma'ayan A. Enrichr: interactive and collaborative HTML5 gene list enrichment analysis tool. *BMC Bioinformatics* **14**: 128 (2013)

11. Laskowski, R., Rullmann, J. A., MacArthur, M., Kaptein, R. & Thornton, J. AQUA and PROCHECK-NMR: Programs for checking the quality of protein structures solved by NMR. *J. Biomol. NMR* **8**, 477–486 (1996).
