# Supplementary material for: The solution structure of Dead End bound to AU-rich RNA reveals an unusual mode of tandem RRM-RNA recognition required for mRNA regulation
Source: Nat Commun. 2022 Oct 6;13:5892. doi: 10.1038/s41467-022-33552-x (PMC9537309; doi:10.1038/s41467-022-33552-x)
Supplement: Supplementary file 3 — Description of Additional Supplementary Files [file 41467_2022_33552_MOESM3_ESM.pdf]

<u>Descriptions of Additional of Supplementary data</u>

Supplementary Data 1 Legend: LC-MS/MS dataset of DND1 co-immunoprecipitations in HEK293T

Supplementary Data 2 Legend: Intra-RNA and Intermolecular restraints used to calculate the Dnd1 RRM12:CUUAUUUG complex ensemble
