# Supplementary material for: The solution structure of Dead End bound to AU-rich RNA reveals an unusual mode of tandem RRM-RNA recognition required for mRNA regulation
Source: Nat Commun. 2022 Oct 6;13:5892. doi: 10.1038/s41467-022-33552-x (PMC9537309; doi:10.1038/s41467-022-33552-x)
Supplement: Supplementary file 5 — Supplementary Data 2 [file 41467_2022_33552_MOESM5_ESM.pdf]

Supplementary Data 2

Related to Figures 3 and 4.

Intra-RNA and Intermolecular restraints used to calculate the Dnd1 RRM12:CUUAUUUG complex ensemble.

Intra-RNA restraints

Res1		Atom1	Res2		Atom2	Distance	Weight
1	RCYT	H6	1	RCYT	H5	2.4	
1	RCYT	H6	1	RCYT	H1'	4.5	
1	RCYT	H6	1	RCYT	H3'	4.5	
1	RCYT	H6	1	RCYT	H2'	3.5	
1	RCYT	H6	1	RCYT	H5'	5	
1	RCYT	H6	1	RCYT	H5"	5	
1	RCYT	H6	1	RCYT	H4'	4.5	
1	RCYT	H5	1	RCYT	H5'	5	
1	RCYT	H5	1	RCYT	H5"	5	
1	RCYT	H5	1	RCYT	H3'	5.5	
1	RCYT	H5	1	RCYT	H2'	5	
1	RCYT	H1'	1	RCYT	H5'	5	
1	RCYT	H1'	1	RCYT	H5"	5	
1	RCYT	H1'	1	RCYT	H4'	4.5	
1	RCYT	H1'	1	RCYT	H2'	4.5	
1	RCYT	H1'	1	RCYT	H3'	4.5	
1	RCYT	H2'	1	RCYT	H5'	5	
1	RCYT	H2'	1	RCYT	H5"	5	
1	RCYT	H2'	1	RCYT	H4'	4.5	
1	RCYT	H3'	1	RCYT	H5'	4.5	
1	RCYT	H3'	1	RCYT	H5"	4.5	
1	RCYT	H3'	1	RCYT	H4'	4.5	
1	RCYT	H3'	1	RCYT	H2'	4.0	
1	RCYT	H4'	1	RCYT	H5'	4	
1	RCYT	H4'	1	RCYT	H5"	4	
1	RCYT	H6	2	URA	H5"	6	
1	RCYT	H1'	2	URA	H5'	6	
1	RCYT	H1'	2	URA	H5"	6	
1	RCYT	H3'	2	URA	H6	5	
1	RCYT	H3'	2	URA	H5	5	
1	RCYT	H3'	2	URA	H1'	6	
1	RCYT	H6	2	URA	H6	5.0	
1	RCYT	H5	2	URA	H5	5.0	
1	RCYT	H1'	2	URA	H6	5.0	
1	RCYT	H4'	2	URA	H5"	5	
2	URA	H6	2	URA	H1'	4.5	
2	URA	H6	2	URA	H2'	4.0	
2	URA	H6	2	URA	H3'	4.5	
2	URA	H6	2	URA	H4'	4.5	
2	URA	H6	2	URA	H5	2.4	
2	URA	H6	2	URA	H5'	4.5	
2	URA	H6	2	URA	H5"	4.5	
2	URA	H5	2	URA	H3'	4.5	
2	URA	H5	2	URA	H2'	4.5	
2	URA	H1'	2	URA	H5'	5	
2	URA	H1'	2	URA	H5"	5	
2	URA	H1'	2	URA	H2'	4.0	
2	URA	H1'	2	URA	H4'	4	
2	URA	H1'	2	URA	H3'	4	
2	URA	H2'	2	URA	H3'	3.5	
2	URA	H3'	2	URA	H5'	4.5	
2	URA	H3'	2	URA	H5"	4.5	
2	URA	H3'	2	URA	H4'	4.5	
2	URA	H3'	2	URA	H5	4.5	
2	URA	H4'	2	URA	H5'	3.5	
2	URA	H4'	2	URA	H5"	3.5	
2	URA	H4'	2	URA	H3'	3.5	
2	URA	H5'	2	URA	H5"	3.5	
3	URA	H6	3	URA	H5'	4.5	
3	URA	H6	3	URA	H5"	5	
3	URA	H6	3	URA	H2'	4	
3	URA	H6	3	URA	H3'	4.5	
3	URA	H6	3	URA	H5	2.4	
3	URA	H6	3	URA	H1'	4.5	
3	URA	H1'	3	URA	H5"	5	
3	URA	H1'	3	URA	H5'	5	
3	URA	H1'	3	URA	H2'	4.0	
3	URA	H1'	3	URA	H4'	4.5	
3	URA	H1'	3	URA	H3'	4.5	
3	URA	H2'	3	URA	H5'	4.5	
3	URA	H2'	3	URA	H5"	4.5	

Supplementary Data 2

3	URA	H3'	3	URA	H5'	4.5	
3	URA	H3'	3	URA	H5"	4.5	
3	URA	H3'	3	URA	H2'	4.0	
3	URA	H3'	3	URA	H4'	4.0	
3	URA	H4'	3	URA	H5'	3.5	
3	URA	H4'	3	URA	H5"	3.5	
3	URA	H1'	4	RADE	H8	5	
3	URA	H3'	4	RADE	H8	5	
3	URA	H4'	4	RADE	H8	5	
3	URA	H4'	4	RADE	H5'	5	
3	URA	H4'	4	RADE	H5"	5	
4	RADE	H2'	3	URA	H1'	5	
4	RADE	H8	4	RADE	H1'	4.5	
4	RADE	H8	4	RADE	H2'	3.5	
4	RADE	H8	4	RADE	H3'	4.5	
4	RADE	H8	4	RADE	H5'	5	
4	RADE	H8	4	RADE	H5"	5	
4	RADE	H1'	4	RADE	H2'	4.5	
4	RADE	H1'	4	RADE	H4'	4.5	
4	RADE	H1'	4	RADE	H3'	4.5	
4	RADE	H3'	4	RADE	H5'	4.5	
4	RADE	H3'	4	RADE	H5"	4.5	
4	RADE	H3'	4	RADE	H4'	4.5	
4	RADE	H3'	4	RADE	H2'	4.0	
4	RADE	H1'	5	URA	H2'	5.0	
4	RADE	H1'	5	URA	H5	3.5	
4	RADE	H1'	5	URA	H6	3.5	
4	RADE	H3'	5	URA	H6	5	
4	RADE	H8	5	URA	H5	5	
5	URA	H6	5	URA	H1'	4.5	
5	URA	H6	5	URA	H5	2.4	
5	URA	H6	5	URA	H2'	3.5	
5	URA	H6	5	URA	H4'	5	
5	URA	H6	5	URA	H3'	4.5	
5	URA	H5	5	URA	H2'	5	
5	URA	H1'	5	URA	H2'	4.0	
5	URA	H1'	5	URA	H3'	4.0	
5	URA	H1'	5	URA	H5	5.5	
5	URA	H2'	6	URA	H5	4.5	
5	URA	H2'	6	URA	H6	4.5	
5	URA	H3'	6	URA	H5	4.5	
6	URA	H6	6	URA	H1'	4.5	
6	URA	H6	6	URA	H5	2.4	
6	URA	H6	6	URA	H2'	4.5	
6	URA	H6	6	URA	H3'	4.5	
6	URA	H6	6	URA	H4'	6	
6	URA	H6	6	URA	H5'	5	
6	URA	H6	6	URA	H5"	6	
6	URA	H5	6	URA	H4'	6	
6	URA	H5	6	URA	H2'	5	
6	URA	H1'	6	URA	H5'	5	
6	URA	H1'	6	URA	H5"	5	
6	URA	H1'	6	URA	H4'	5	
6	URA	H1'	6	URA	H3'	5	
6	URA	H1'	6	URA	H2'	4.5	
6	URA	H2'	6	URA	H5	4.5	
5	URA	H3'	6	URA	H5'	4.5	
5	URA	H3'	6	URA	H5"	4.5	
6	URA	H1'	7	URA	H5"	5.5	
7	URA	H6	7	URA	H2'	4	
7	URA	H6	6	URA	H3'	5	
7	URA	H6	7	URA	H5	2.4	
7	URA	H6	7	URA	H1'	4.5	
7	URA	H5	7	URA	H2'	5	
7	URA	H5	6	URA	H3'	5.5	
7	URA	H1'	7	URA	H2'	4.5	
7	URA	H1'	7	URA	H5	4.5	2
7	URA	H2'	7	URA	H5'	5	
7	URA	H2'	7	URA	H5"	4.5	
7	URA	H2'	8	RGUA	H8	6	
7	URA	H6	8	RGUA	H1'	4.5	
8	RGUA	H8	8	RGUA	H1'	4.5	
8	RGUA	H8	8	RGUA	H3'	4	
8	RGUA	H8	8	RGUA	H4'	5	
8	RGUA	H8	7	URA	H4'	6	
8	RGUA	H1'	8	RGUA	H5"	5	
8	RGUA	H1'	8	RGUA	H5'	5	

Supplementary Data 2

Intermolecular restraints

Res1		Atom1	Res2		Atom2	Distance
1	RCYT	H1'	130	LEU	QD1	5
2	URA	H6	130	LEU	QD1	5
3	URA	H6	130	LEU	QD1	5
2	URA	H6	130	LEU	QD2	5
3	URA	H6	130	LEU	QD2	5
2	URA	H5	130	LEU	QD1	6
2	URA	H5	130	LEU	QD2	6
2	URA	H1'	130	LEU	QD1	5
3	URA	H5	130	LEU	QD1	5
2	URA	H1'	130	LEU	QD2	5
3	URA	H5	130	LEU	QD2	5
2	URA	H2'	130	LEU	QD1	4
1	RCYT	H2'	130	LEU	QD1	4
2	URA	H2'	130	LEU	QD2	4
2	URA	H2'	130	LEU	HG	5
1	RCYT	H2'	130	LEU	QD2	4
2	URA	H3'	130	LEU	QD1	5
2	URA	H3'	130	LEU	QD2	6
3	URA	H1'	130	LEU	QD1	5
3	URA	H1'	130	LEU	QD2	5
3	URA	H4'	130	LEU	QD2	5
3	URA	H4'	130	LEU	QD1	5
3	URA	H1'	61	PHE	HZ	6
3	URA	H5'	64	ARG	QG	5
3	URA	H5"	64	ARG	QG	6
3	URA	H5"	64	ARG	QD	6
4	RADE	H2	135	THR	QG2	5
4	RADE	H2	135	THR	HB	6
4	RADE	H2	90	MET	QE	6
4	RADE	H2	61	PHE	QD	6
4	RADE	H2	102	TYR	QE	3.5
4	RADE	H2	102	TYR	QD	4
4	RADE	H8	61	PHE	HZ	6
4	RADE	H8	61	PHE	QE	6
4	RADE	H1'	61	PHE	QE	6
4	RADE	H1'	61	PHE	HZ	6
4	RADE	H1'	100	PHE	QD	6
4	RADE	H1'	100	PHE	QE	6
4	RADE	H2'	100	PHE	QE	6
4	RADE	H4'	100	PHE	QE	5
4	RADE	H5"	100	PHE	QE	5
4	RADE	H5"	100	PHE	QE	5
4	RADE	H1'	90	MET	QE	5
4	RADE	H3'	100	PHE	QE	5
5	URA	H6	137	LYS	QD	4
5	URA	H5	137	LYS	QD	5
5	URA	H1'	137	LYS	QD	6
5	URA	H6	137	LYS	QG	6
5	URA	H5	137	LYS	QG	6
5	URA	H5	135	THR	QG2	6
5	URA	H6	90	MET	QE	5
5	URA	H1'	102	TYR	QR	4.5
5	URA	H4'	102	TYR	QR	5
5	URA	H1'	100	PHE	QE	4.5
5	URA	H1'	189	HIS	HE1	6
5	URA	H4'	100	PHE	QE	4.5
5	URA	H2'	100	PHE	QE	5.5
5	URA	H4'	90	MET	QE	5
5	URA	H2'	90	MET	QE	5
5	URA	H1'	90	MET	QE	3.5
6	URA	H3'	36	VAL	QG1	6
6	URA	H6	193	ALA	QB	5
6	URA	H5	193	ALA	QB	4.5
6	URA	H1'	193	ALA	QB	3.5
6	URA	H5	215	TRP	HH2	4
6	URA	H6	215	TRP	HH2	6
6	URA	H1'	197	LYS	HG3	5
6	URA	H1'	197	LYS	HG2	5
6	URA	H1'	197	LYS	HD3	4
6	URA	H1'	197	LYS	HD2	4
6	URA	H1'	197	LYS	QB	4
6	URA	H1'	197	LYS	QE	3.5
6	URA	H1'	197	LYS	HA	6
6	URA	H1'	194	MET	QE	3.5
6	URA	H4'	194	MET	QE	4
6	URA	H1'	194	MET	HA	6
6	URA	H2'	197	LYS	QE	5
6	URA	H4'	197	LYS	QE	4
7	URA	H4'	36	VAL	QG1	5

Supplementary Data 2

7	URA	H3'	37	ASN	HD21	5
7	URA	H5'	36	VAL	QG1	5
7	URA	H5'	39	GLN	QG	5
7	URA	H5'	36	VAL	HB	6
7	URA	H5	92	THR	QG2	5
7	URA	H6	92	THR	QG2	5
7	URA	H4'	197	LYS	QE	6
7	URA	H5'	197	LYS	QE	6
7	URA	H5"	197	LYS	QE	6
8	RGUA	H8	93	PHE	QE	5
8	RGUA	H8	92	THR	QG2	6.0
8	RGUA	H1'	92	THR	QG2	5.0
8	RGUA	H3'	92	THR	QG2	6.0
8	RGUA	H3'	93	PHE	QE	5
